# Nanoparticles as an alternative treatment for bovine mastitis -A review

**DOI:** 10.5713/ab.24.0590

**Published:** 2025-02-27

**Authors:** Beatriz Elena Castro-Valenzuela, Moisés Armides Franco-Molina, Cristina Rodríguez-Padilla

**Affiliations:** 1 Universidad Autónoma de Nuevo Léon, Facultad de Ciencias Biológicas, Laboratorio de Inmunología y Virología, San Nicolás de los Garza, Nuevo León, México

**Keywords:** Antibacterial, Bovine Mastitis, Dairy Cattle, Nanoparticles, Nanotechnology

## Abstract

Nanotechnology is causing revolutionary changes in different disciplines such as food, pharmacology, medical, and agriculture. Nanotechnology allows for the development of materials or devices with unique physical, chemical, and biological properties that differ from their precursor materials, such as nanoparticles. These have gained significant relevance in veterinary medicine, with the potential to be the future treatment for bovine mastitis. Losses in the global dairy industry of up to USD 35 billion annually caused by this pathology have been determined. Despite advances in the genetic selection of dairy cattle and management practices, the control of bovine mastitis remains inadequate. Antibiotics have been the most prevalent treatment for this condition. However, the residues of the antibiotics in milk compromises its quality. Furthermore, the emergence of bacterial resistance has necessitated the search for alternative strategies for treating mammary gland infections, opening a field of opportunities for the nanoparticle-based therapy. This review addresses key topics to elucidate the potential use of these nanoparticles as a treatment strategy for bovine mastitis.

## INTRODUCTION

Mastitis is the most prevalent, significant, and costly disease of dairy farms since it represents substantial economic losses by causing a reduction in milk yield and quality, premature slaughter and replacements of animals, and costs in veterinary services and drugs [1]. Furthermore, it can cause reduced fertility and metabolic dysfunctions in affected animals. Total annual economic losses from mastitis worldwide have been estimated at USD 35 billion [2]. Despite implementing management practices and advances in the genetic selection of dairy cattle, the control of bovine mastitis remains inadequate [3,4]. Mastitis is a pathology characterized by inflammation in the mammary gland parenchyma, the most common cause being infectious agents [5]. The main pathogens causing mastitis are *Staphylococcus aureus*, *Streptococcus* spp., and *Escherichia coli* [6,7]. Currently, the most common therapy used for its control is antibiotics, however, the presence of residues of these drugs in milk, the emergence of bacterial resistance [8–10], and the constant concern about the spreading of these strains to humans, make it necessary to search for alternative strategies to prevent and eliminate infections in the mammary gland. Through nanotechnology, it is possible to develop materials at the nanometric level with unique physical, chemical, and biological properties, different from those of their precursor materials, such as nanoparticles (NPs). The NPs have a greater area: volume ratio and have unique chemical and physical properties, its physical properties permit them to be promising antimicrobial agents. Therefore, NPs are currently of great interest due to the appearance of an increasing number of multidrug pathogens resistant to traditional antibiotics. Nanometric-sized particles are applied in human, veterinary, pharmaceutical medicine, and other disciplines with multiple applications [11]. Nowadays, metallic, and polymeric NPs, alone or combined have been evaluated: Pt, Ag, Au, Cu, ZnO, Cu/Ag, Cu/CuO, and chitosan [12–18], at least *in vitro*, for combated the bovine mastitis. Nonetheless, even though promising results have been obtained, it is necessary to develop low-cost alternatives within the reach of any dairy producer. This review addressed topics of interest to elucidate the potential use of these NPs as both a preventive method and a treatment for bovine mastitis.

## GLOBAL AND ECONOMIC IMPACT OF THE DAIRY INDUSTRY

The world population is increasing constantly, according to the FAO it is expected that by the year 2050, there will be 9.73 billion inhabitants in the world. This represents a significant challenge for agricultural and food systems, which must increase their production by 50% compared to 2013 [19] to meet the growing food demands of the population. Therefore, it is necessary to optimize the efficiency of the agricultural sector.

Among the economic activities of the primary sector, milk production stands out due to its faster growth rate compared to other major agricultural products. Milk production occurs worldwide and has increased by more than 60%, rising from 530 million tons (Mt) in 1988 to 852 Mt in 2019. This growth is attributed to factors such as population growth, increased income, urbanization, and changes in diet in countries like China and India [20]. Also, it is forecast to grow by 1.6% annually between 2020 and 2029. Therefore, it is estimated that by 2029, global milk production will reach 997 Mt, with bovine cattle contributing 81% of this production [21]. More than 80% of the world’s population, or about 6 billion people, regularly consume liquid milk or other dairy products, which are trusted sources of nutrition and commodities worldwide [20].

## ECONOMIC IMPACT OF MASTITIS

The productivity of the dairy cow is influenced by a wide range of factors, including climatic variations, changes in nutritional management, bromatological factors, health, and reproductive value, as well as the animal’s genetic potential. Among these factors, health greatly impacts production costs [22]. Within this category, mastitis is particularly notable as one of the most prevalent, significant, and costly diseases affecting the dairy industry [12].

Mastitis is considered the disease that represents the most economic losses in the dairy sector since it reduces milk yield and quality, obtaining milk unsuitable for sale (discarded milk), premature slaughter of animals and replacements, and expensive service veterinarians and drugs [16,23]. Additionally, cows with mastitis can have reduced fertility and metabolic dysfunctions, which causes important losses in production costs.

A study conducted in China from 2015 to 2017, found that the monthly economic losses associated with clinical mastitis ranged from 12,000 to 76,000 US dollars per farm [24]. In the U.S.A., it is estimated that these losses amount to up to 2 trillion dollars annually, while globally, direct losses are estimated to reach 35 trillion US dollars in direct losses [2].

## BOVINE MASTITIS PATHOLOGY

Mastitis is a disease characterized by infection and inflammation of the mammary gland caused by multiple factors, which can be classified into two groups: the first contemplates microorganisms and the second incorrectly applied techniques during milking, metabolic disorders, udder injuries, and various stress factors [25]. This is because the teat orifice is the first line of defense against the invasion of mastitis-causing pathogens into the teat canal and mammary gland, so the presence of high bacterial contamination increases the opportunity for entry of more bacteria into the teat orifice and causes infections [26]. Mastitis caused by potentially pathogenic microorganisms is more common [27] because milk serves as an excellent culture medium for many of these pathogens. Regardless of the cause, mastitis is characterized by damage to the parenchymal tissue of the udder, leading to a reduction in the number and activity of epithelial cells and, consequently, a decrease in milk production [28].

### Classification of mastitis

Mastitis is generally classified into clinical and subclinical forms, depending on the severity of the infection [29]. The severity is influenced by factors such as the nature of the causal pathogen, the age, and breed of the animal, it is health status, immunological condition, and stage of lactation [30].

Clinical mastitis is easily detected since it is characterized by the presence of physiological symptoms such as loss of appetite, fever, dehydration, or depression; and alterations in the udder (inflammation, swelling, etc.), as well as in the milk (presence of clots, scales, watery appearance, viscosity, etc.) [29].

Unlike clinical mastitis, subclinical mastitis is not easily detected since there are no apparent physical changes in the mammary gland or the milk. Therefore, tests are necessary to identify indicators of inflammation in the mammary gland for diagnosis of the type of mastitis [30]. This condition is characterized by an increased somatic cell count (SCC); mainly due to leukocytes. Consequently, the traditional method for detecting inflammation in the udder involves counting these cells [29].

### Traditional methods bovine mastitis detection

SCC is considered a universal detection indicator for mastitis and is widely used to determine milk quality in individual quarters of the udder [31], due to its cost and easy data collection [25]. The somatic cells present in the milk of healthy cows are mainly macrophages (66% to 88%), in addition to neutrophils, epithelial and mononuclear cells. The proportion of neutrophils is only 1% to 11% in a healthy quarter but increases to 90% or more in an intramammary infected (IMI) quarter, SCC has been extensively used as an indicator of IMI since 1960. The limit of SCC in a healthy quarter is 200,000 cells/mL [31].

SCC can also be estimated using the California Mastitis Test (CMT), an indirect assay based on the DNA content of milk. This test consists of a detergent that contains bromocresol blue, which is used to lyse the plasma membrane of somatic cells; consequently, DNA and cellular proteins are released and precipitate, forming a viscous gel-like matrix when added to milk [25]. The viscosity is proportional to the number of leukocytes present in the sample. The advantages of this test are that it is inexpensive, fast, and can be done in the field, near the animal analyzed [30]. Thus, a cow is diagnosed with clinical mastitis if it presents at least a quarter of the udder with modified appearance milk: with clots or scales or milk with a watery appearance. A cow is diagnosed with subclinical mastitis if the last SCC is greater than 200,000 cells per mL or if the CMT was positive on the day of sampling, despite a normal appearance of the milk [32].

### Most frequent pathogens involved in mastitis

Mastitis induces permanent and irreversible damage to milk-producing glandular tissue [28]. Today, 137 different microorganisms have been identified as causing bovine mastitis, including bacteria, viruses, yeasts, and algae, with bacteria being the main causative agents of the disease (95%). The bacteria that cause the most common forms of mastitis are *Staphylococcus aureus*, *Streptococcus agalactiae*, *Streptococcus dysgalactiae*, *Streptococcus uberis*, *E. coli*, *Mycoplasma* spp. and *Klebsiella* spp. [4,33,34].

#### Biofilm formation, virulence factor**:**

Microorganisms possess virulence factors that enable them to survive under adverse conditions, one of which is the formation of biofilms. A biofilm is a multicellular structure with a specific composition, consisting of water and extracellular polymeric substances, mainly made up of polysaccharides, proteins, nucleic acids, and surfactants. This structure allows microorganisms to adhere strongly to both biotic and abiotic surfaces, protecting against environmental damage. The exopolysaccharide matrix may also be impermeable to antimicrobials, inhibiting their penetration into the biofilm [35].

Biofilms are groups of microorganisms embedded in an extracellular matrix of polysaccharides produced by the microorganisms themselves. This matrix is irreversibly associated with the surface to which it is attached, which may include host tissue. This leads to chronic infections, as the biofilm protects bacteria from antibiotics and makes the microorganisms more effective at invading mammary epithelial cells [28].

## TECHNIQUES FOR THE CONTROL OF BOVINE MASTITIS

Despite implementing management practices and advances in the genetic selection of dairy cattle, the control of bovine mastitis remains inadequate. Antibiotics are currently the most effective strategy for treating and preventing future IMIs. However, the misuse and overuse of antibiotics in managing bovine mastitis have led to the development of resistant pathogens [16]. As a result, this antibiotic-centered approach has notable drawbacks, including increased antibiotic resistance, low infection clearance rates, and the presence of antibiotic residues in milk. According to a report by the World Health Organization, bacterial resistance to antibiotics poses a global threat to public health, ranking alongside climate change and terrorism. This underscores the urgent need for the development of new, safe, and cost-effective antibiotics [13]. Additionally, antibiotic resistance in microorganisms can be transmitted from animals to humans through the food chain, raising potential concerns for human health.

### Management of dairy cattle in the dry period (intramammary sealant)

Management of the dairy cow in the dry period is crucial for the health of the udder since it is a period where the ideal conditions for the regeneration of the mammary tissue are created [36].

At the end of a milk production cycle, the epithelium of the cow’s teat duct produces keratin that physically traps bacteria and blocks their migration to the mammary gland cistern. This barrier produced naturally by epithelium also has antimicrobial activity due to the bacteriostatic fatty acids (lauric, myristic, palmitoleic, and linoleic), and the fibrous proteins it possesses [37]. The nipple orifice and duct are considered the most important physical barriers of the defense system against the penetration of microorganisms into the mammary gland. Despite this, the time it takes the teat canal to close varies between animals. In one study, it was shown that 50% of the teat canals closed 7 days after drying off, 45% closed within the following 50 to 60 days of the dry period, and the remaining 5% did not close until after 90 days of drying off [38]. Due to this, in dairy cattle production systems, the use of intramammary internal sealants has been implemented, which currently has bismuth subnitrate as its active principle, since this compound is biologically inert [39], acts as a physical barrier in the teat canal of dry cows and has antibacterial activity [40]. Additionally, the use of internal sealants at dry-off has been shown to significantly reduce the incidence of IMIs and clinical mastitis in dairy cows by up to 23% [41,42].

Despite the successful results reported, the dairy industry still needs to improve these parameters. For this reason, conventional dry-off sealants (bismuth subnitrate) combined with commercial antibiotics have been used to prevent the occurrence of mastitis. The results obtained with this combination have proven to be more effective for preventing mastitis than when applied separately [43].

The emergence of multidrug-resistant strains has taken place worldwide, depending on the region, strains resistant to certain antibiotics have been found. In Mexico, the bovine mastitis isolates showed a resistance pattern to beta-lactam antibiotics, and protein synthesis inhibitors mainly penicillin, clindamycin, and cefotaxime [44]. In Taiwan, *E. coli* isolates from the cases of clinical mastitis showed resistance to cloxacillin, tetracycline, neomycin, gentamycin, ampicillin, ceftriaxone, cefotaxime, and ceftazidime [45]; in some European countries, the highest antimicrobial resistance was observed against benzylpenicillin and oxytetracycline [46]. Isolated bacteria causing mastitis resistant to penicillin, amoxicillin, oxytetracycline, and methicillin have been found in Asia [47]. This shows that resistance can be generated to almost any type of antibiotic and, although its use has not been banned, strategies have been developed to reduce their usage. One of them is the Alliance for the Responsible Use of Medicines in Agriculture (RUMA) of the United Kingdom, an intersectoral organization that aims to reduce or refine antimicrobials in animal agriculture, due to the resistance generated to these drugs by their excessive and indiscriminate use [48].

## RESISTANCE OF PATHOGENS TO MASTITIS TREATMENT

Nowadays, several strains of multidrug-resistant bacteria have been isolated from cases of bovine mastitis such as coagulase-negative *Staphylococcus* (CNS) [9,49]; *Streptococcus dysgalactiae* [50]; *Streptococcus uberis* [9,51]; *E. coli* [9,52]; *Staphylococcus aureus* and *Streptococcus agalactiae* [8,27,49,53]. All these strains can cause severe infections, but in the case of *S. aureus*, this is due to its ability to form biofilm on the surfaces of its host, since it is also an evasion mechanism for the immune system [54]. This demonstrates the constant increase in strains resistant to drugs involved in causing this pathology.

## ALTERNATIVE TREATMENTS AGAINST MASTITIS

Pathogens have developed mechanisms to persist in the udder, such as biofilm formation and internalization in bovine epithelial cells [13]. Due to the emergence of multidrug-resistant strains of mastitis-causing pathogens, new therapies are needed to reduce or replace antibiotic therapies. Some of them are the use of plant extracts with antimicrobial activity, immunotherapy, vaccines, supplementation with vitamins and minerals, probiotics, management practices in the dry period of the dairy cow, bacteriophages [55], and NPs [56–58].

Based on the microorganisms identified as causes of mastitis, various vaccines have been developed to protect against one or more pathogens. Although these vaccines are commercially available (Lysigin, Startvac, Mastivac, etc.) their effectiveness remains variable and inconsistent [59–64], due to the vaccination may improve health by reducing the severity of infection, but it does not prevent the disease effectively (Table 1).

Besides antibiotics and vaccines nowadays there are other strategies to combat bovine mastitis, such as plant-derived compounds, probiotics, and bacteriophages. In the case of plant-derive, different compounds have been evaluated, such as oil essentials (rosemary, *Thymus serpyllum*, *Thymus vulgaris*) or extracted medicinal plants and exhibited antimicrobial efficacy, as well as limited or no side effects compared to conventional drugs [72,73]. However, although several scientific papers discuss the potential of natural compounds as alternatives against infections in animal production, only a limited number of studies provide robust scientific evidence, due to the use of plant-derived, they have some disadvantages. Toxicologic effects need to be studied given the complexity of their composition; the establishment of a standardized extraction process is necessary because their biological activity is strongly associated with phases of the process extraction, type of solvent, storage conditions, final concentration of active compounds, and other factors that can cause variations between in the effectiveness of the extract [73].

Probiotic microorganisms have demonstrated prevention of mastitis, thanks to induced inhibition and antagonistic activity against pathogens causing mastitis, such as *S. aureus*. Because can interact with pathogens, adhere to epithelial cells, auto-aggregation, and co-aggregation pathogens, produce antagonistic metabolites, compete for nutrients, and have immunomodulatory activity [59]. Nevertheless, although some reports show the benefits of their administration locally, it is necessary to determine the best route of administration, and maintaining the adequate concentration of probiotics to achieve their effectiveness is expensive [74].

Lytic activity of bacteriophages against pathogens causing bovine mastitis has been evaluated, at least, *in vitro* methods demonstrating that the phages possess bactericidal activity [75,76]. Nonetheless, some studies showed significant degradation or inactivation of the phages when they are inside the mammary gland [77]. Therefore, more *in vivo* studies must be realized to demonstrate the efficacy of bacteriophages in therapeutic applications; as well as to determine an adequate volume and concentration for their administration. On the other hand, is essential to evaluate the cost-benefit of the use of this alternative therapy.

In the context of alternative strategies to combat mastitis, the use of NPs is the most promising option, due to their excellent bactericidal effect and the low cost involved in their production. The NPs have a higher area:volume ratio and unique chemical and physical properties that allow them to penetrate deeper into the interior of cellular structures freely passing through the barriers of organisms and besides, whereby both polymeric and metallic NPs exhibit potential as antimicrobial agents, at least *in vitro*. Additionally, to the fact that the concentrations necessary to achieve this effect are very low [13,15,17,78]. However, the safety of these materials in living organisms still needs to be confirmed to ensure that their use does not result in unwanted effects, such as cytotoxicity in healthy tissues. Evaluations on the use of NPs *in vivo* models are very limited. Currently, there is only one study where the use of metallic NPs on mastitis was assessed in a murine model (Table 2).

## NANOPARTICLES IN THE TREATMENT OF MASTITIS

### Polymeric nanoparticles

Some experiments have been carried out, at least *in vitro*, to evaluate the effect of chitosan on mastitis-causing pathogens, in one of them it was found that chitosan has antimicrobial activity against different strains of *Staphylococcus aureus* and CNS isolated from cases of bovine mastitis, but when it is administered as a NP (138 nm of diameter) this activity increases. The minimum inhibitory concentration (MIC) values of the NPs were found in a range of 200 to 400 μg/mL for *S. aureus* and 400 to 800 μg/mL for CNS. The minimum bactericidal concentration of the NPs was 400 μg/mL for all *S. aureus* strains and 800 μg/mL for most CNS strains. This study showed that chitosan NPs cause damage to the plasma membrane of the bacterium causing lysis. Additionally, these NPs can inhibit the formation of biofilms of the bacteria, and the effective concentration with antibacterial activity (12.5 μg/mL) is not toxic for bovine cell lines [14].

Another investigation demonstrated that the bactericidal effect of chitosan NPs on pathogens causing bovine mastitis and their ability to inhibit biofilm formation depends on the size of the NPs, the smaller the NPs are more efficient. In addition, it was observed that NPs are capable of internalizing in *S. aureus* and do not promote the production of proinflammatory cytokines. Furthermore, it has also been shown that chitosan NPs with a spherical shape of 19.1 nm in diameter obtained by ionic gelation have antimicrobial activity against *Pseudomonas sp*. isolated from a case of bovine mastitis infection, as well as they are capable of inhibiting biofilm formation in an initial stage and dispersing the stability of the mature biofilm [16].

### Metal nanoparticles

#### Mechanisms of action of metal nanoparticles against mastitis

The mechanism of action of NPs as antimicrobial agents is similar to that of commercial antibiotics, meaning they can act through one or more mechanisms to inhibit the growth or kill invading organisms [79,80]. These mechanisms focus mainly on molecular aspects, including damage to molecules such as DNA, proteins, and cell membranes, physical disruption of cell structures (such as the cell wall, plasma membrane, ribosomes, etc.), as well as the generation of new molecules, like reactive oxygen species (ROS) and alteration in signal transduction [81,82].

The mechanisms of action of NPs include disruption of the cell membrane, disruption of the electron transport chain, ROS production, and damage to the pump of proton efflux, all contribute to damage in the cytoplasm, plasmid, DNA content, and protein synthesis [87].

Nanomaterials have an advantage over other antibacterial molecules in attacking microorganisms capable of forming biofilms because their surface area greatly exceeds their size, and they can easily penetrate this structure. One pathway by which biofilm production is inhibited is through inhibition of Quorum sensing signaling, where in Gram-negative bacteria, small diffusible autoinducers such as N-acyl homoserine lactones combine with related receptors to produce complexes that act as signaling molecules and are identified by intracellular kinases, in the case of Gram-positive bacteria, modified oligopeptides or self-induced peptides act as said molecules. These complexes activate phosphorylation cascades to induce phosphorylation of a response regulatory protein. In both cases, these complexes bind to a DNA promoter region responsible for activating transcription factors of certain virulence factors, such as the genes necessary for biofilm production. The inhibition of “quorum sensing” involves the inactivation of molecular signals for the formation of bacterial biofilm [94,95].

Another mechanism of action is that the ROS produced by metallic NPs can directly damage constituents within the bacterium’s cytoplasm, such as causing DNA damage in the nucleolus. DNA damage can also occur due to the release of metallic ions, which disrupt hydrogen bonding between antiparallel polynucleotide strands. Additionally, DNA denaturation can result from intercalation between purine and pyrimidine bases [82]. Furthermore, NPs have been found to dephosphorylate tyrosine residues, leading to inhibition of signal transduction inside the cell.

A particular mechanism specific to metallic NPs is the release of ions. For example, in the case of silver (Ag), some reports indicate that spherical NPs exhibit greater antimicrobial activity than other shapes, such as discoidal or triangular forms. This activity increases since spherical NPs have a larger surface area facilitating the release of more Ag ions, thereby enhancing antimicrobial efficacy [96].

#### In vitro effect of metallic nanoparticles against bovine mastitis

Several metallic NPs have been evaluated for their antimicrobial activity against mastitis-causing pathogens. For example, zinc oxide NPs (ZnO NPs) synthesized by self-combustion reaction with a diameter of 174 nm were tested using the plate diffusion method. It was observed that these NPs could generate growth inhibition halos of bacteria isolated from clinical mastitis cases, such as *E. coli* and *K. pneumoniae*, but did not exhibit any bactericidal effect on *S. aureus*. The probable action mechanism of the ZnO NPs is due to the release of metal ions in solutions that are exposed to bacterial cells, which can penetrate the cell wall and cause toxicity to the bacteria. Moreover, ZnO NPs can induce the production of ROS and cause membrane alterations [13].

The biocidal activity of commercially available gold (Au), silver (Ag), copper (Cu), and platinum (Pt) NPs have also been evaluated by turbidimetry on mastitis-causing pathogenic microorganisms such as *E. coli*, *Streptococcus uberis*, *S. aureus*, *Candida albicans*, and *Candida krusei*. The NPs with the highest cytotoxicity were Ag and Cu, while Au NPs exhibited weak activity, and Pt NPs had no biocidal effect [12]. These results can be because NPs exert their effect simultaneously against different structures and pathways, unlike antibiotics with only specific sites of action.

To increase the bactericidal effect of NPs, NPs of various metals have been studied in combination, and to reduce the risk of toxicity towards eukaryotic cells, NPs synthesized with plant extracts have also been evaluated. For this reason, the biocidal effect of AgNPs and CuNPs independently and combined on the microorganisms *S. aureus* and *E. coli*, the main bacteria involved in inflammation of the mammary gland in bovines, has been determined *in vitro*, and it was found that these NPs can reduce the viability of these pathogens and that in combination their effect is enhanced. In addition, these particles did not have a cytotoxic effect on mammary gland tissue cell lines [15]. Additionally, it has been found that AgNPs and CuNPs evaluated individually and in combination inhibit the formation of biofilms of bacteria causing mastitis: *Streptococcus agalactiae*, *Streptococcus dysgalactiae*, *Salmonella* spp., *Enterococcus faecalis*, *Enterobacter cloacae*, *Candida albicans*, *E. coli*, and *S. aureus*. Moreover, the combination of NPs was the most effective, reducing the biofilm by 100% at a concentration of 200 ppm and MIC of 12.5 ppm for *S. aureus*, 3.125 ppm for *S. agalactiae* and 6.5 ppm for all other microorganisms [17]. The Ag-Cu NPs complex caused the greatest biofilm reduction. These NPs or their agglomerates are too large to penetrate the biofilm, but they can interact with planktonic cells to prevent this spread and reduce biofilm formation [15,17].

AgNPs can inhibit the formation of biofilms by *S. aureus* and *E. coli* bacteria. In the case of *E. coli*, the AgNPs have been seen to be capable of decreasing the transcriptional activity of the genes that code for biofilm formation: bcs A, csgA, fliC, motA, wcaF, fimA. It has also been shown that when combining antibiotic treatments capable of inhibiting protein synthesis with AgNPs, they have a synergistic effect against *S. aureus* isolated from cases of bovine mastitis [81].

Another interesting experiment determined that copper oxide (CuO) NPs synthesized with ginger extract as reducer agent (sizes 23.38 and 46.64 nm) and NPs synthesized with garlic extract as reducer agent (sizes 26 to 56 nm) exhibit antimicrobial activity against multidrug-resistant *S. aureus*. These NPs produce growth inhibition halos of this bacterium ranging from 2.05 to 5.65 mm in diameter for ginger NPs and 1.25 to 11 mm in diameter for garlic NPs. The effect of CuO NPs involves the production of ROS, leading to bacterial lysis [18].

Iron oxide NPs have been incorporated into MALDI-TOF detection systems for the diagnosis of bovine mastitis [97]. Furthermore, they have been explored as an alternative treatment for several bacterial strains associated with mastitis infections [91]. However, the results have been controversial. Studies conducted on Gram-positive and Gram-negative bacteria have reported that iron oxide NPs do not inhibit bacterial growth and may interfere with the antibacterial effect of ciprofloxacin [92].

On the other hand, alternative synthesis methods for iron oxide NPs have shown promising results, including the inhibition of biofilm formation by *Staphylococcus aureus* [93]. Additionally, other reports have demonstrated that iron oxide NPs exhibit a high inhibitory effect on *Bacillus subtilis* and *Bacillus licheniformis*, a moderate effect on *Staphylococcus aureus*, *Bacillus brevis*, *Vibrio cholerae*, *Streptococcus aureus*, *Staphylococcus epidermidis*, and *E. coli*, but no inhibitory effect on *Shigella flexneri* and *Pseudomonas aeruginosa* [91].

However, not all the results obtained with metallic NPs have been successful. For instance, is the case where AgNPs showed reasonable antimicrobial activity (MIC≤16 μg/mL) and inhibition of biofilm formation at concentration of 64 μg/mL against multidrug-resistant *Streptococcus agalactiae* isolated from mastitis cases, but when comparing these results against cinnamon oil, cinnamon alone had a better effect (MIC≤ 0.063 μg/mL and inhibition of biofilm formation of 4 μg/mL) than even in combination with NPs [15].

Another discouraging result was the finding that *S. aureus* can develop resistance to the antimicrobial effect of AuNPs and AgNPs. Also, these NPs exhibited cytotoxic effects in Wistar rats. In this experiment, it was found that the MIC of AgNPs with sizes of 10 and 20 nm, and AuNPs with diameters of 10 and 20 nm for 198 different strains of *S. aureus* was 14.70±1.19 μg/mL, 9.15±0.13 μg/mL, 24.06±2.36 μg/mL, and 18.52±1.26 μg/mL, respectively. However, most of the strains developed strong resistance to AgNPs treatment, whereas only two strains were resistant to 10 nm AuNPs and three strains to 20 nm AuNPs. Organ histopathology revealed that the 2 mg/kg dose of NPs damaged the brain, liver, kidney, heart, spleen, and lung. Thus, it was found that *S. aureus* strains could develop resistance less frequently against AuNPs than AgNPs and that they are toxic in rats at a dose of 2 mg/kg [79].

But what information is available regarding the bismuth NPs? Despite the promising results on antimicrobial activity with this element, and the known enhancement of activity when bismuth (Bi) is administered in NP form [98], such NPs have not been evaluated for preventing or combating bovine mastitis. However, the antimicrobial activity of Bi NPs has been tested *in vitro* against microorganisms including *Campylobacter jejuni*, *Listeria monocytogenes*, *Yersinia enterocolitica*, *Salmonella typhimurium*, *E. coli*, *Mycoplasma argini*, *Acholeplasma ladidlawii*, *Bacillus anthracis*, *Leptospira Pomona* [85], *S. aureus*, and *Candida albicans* [86], some of which could be causative agents of bovine mastitis. Additionally, Bi NPs have been shown to reduce microbial biofilm formation [87-89] and exhibit antiviral activity [58].

In 2019, Martínez *et al*., demonstrated that chitosan-based membranes supplemented with lipophilic Bi NPs caused complete inhibition of biofilm formation and 90% to 98% growth inhibition of six different oral pathogens: *Poryphyromonas gingivalis*, Methicillin-resistant *S. aureus*, *Candida albicans*, *E. coli*, *Enterococcus faecalis*, and *Streptococcus gordonii*.

Finally, Bi NPs reduced with *Moringa oleifera* leaf extract with a diameter of 40.4 to 57.8 nm were evaluated and it was found that the MIC for *E. coli* and *S. aureus* was 500 μg/mL [90].

#### In vivo effect of metallic nanoparticles against bovine mastitis

The toxicity of some metallic NPs has been evaluated, but only in short-term experiments in cell cultures. However, the results of other studies have not been reassuring [79,98]. Therefore, it is necessary to carry out more experiments to demonstrate the safety of the application of NPs in living organisms.

Only one type of metallic NPs has been evaluated *in vivo*, largely due to the difficulties associated with working with large ruminants like cattle, which involved significant economic implications for maintaining experimental units. Some researchers argue that murine models are not suitable for studying bovine mastitis due to the physiological differences between these species. After lactation ends, the mammary gland undergoes involution; in mice, this process involves extensive degeneration of mammary epithelial or alveolar cells, leading to a primary ductal morphology [99]. In contrast, cattle experience minimal cell loss during post-lactation involution [100].

However, recently a study was conducted in which mastitis caused by *S. aureus* was induced in Wistar rats, which were then treated with CuNPs and compared with rats treated intramuscularly with conventional antibiotic (gentamicin). The results were encouraging. Administration of CuNPs at a concentration of 6.25 μg/mL produced a significant zone of inhibition in an *in vitro* sensitivity test and showed minimal toxicity in fibroblast cell lines. Clinical signs in the CuNPs-treated group improved within three days of treatment, whereas improvement in the gentamicin-treated group, was observed by the 4th day. On the 5th day of treatment, the bacterial load, mammary glands weight, total oxidative state, and oxidative stress index were significantly lower in the CuNPs group compared to the commercial antibiotic group. Additionally, CuNPs treatment showed a marked improvement in histopathological changes compared to the gentamicin-treatment group. Therefore, it was concluded that CuNPs could offer a potential alternative therapeutic regimen for the treatment of mastitis in bovines [78].

Despite the limited *in vivo* studies on the administration of metallic NPs, it is known that some medical devices containing AgNPs can release silver ions into the bloodstream, where they accumulate in organs such as the liver, spleen, and kidney. This accumulation can lead to severe toxicity and potentially death [83]. In rats treated with AgNPs, these NPs can accumulate in specific tissues, including the spleen, liver, lungs, and kidneys, where Ag ions may be generated. These ions can cause chromosome damage, suggesting possible genotoxicity associated with AgNPs [84].

## CONCLUSION

Mastitis represents one of the main challenges for the dairy industry, and despite various techniques implemented to combat it, they have often proven inefficient. Currently, bovine mastitis is commonly treated with antibiotics. However, this approach faces a major drawback: the emergence of multidrug-resistant pathogen strains. Therefore, developing new, safe, and cost-effective treatments is crucial. In this context, both polymeric and metallic NPs exhibit potential as antimicrobial agents, at least *in vitro*. However, the safety of these materials in living organisms still needs to be confirmed to ensure that their use does not result in unwanted effects, such as cytotoxicity in healthy tissues.

## Figures and Tables

**Table 1:** Effectiveness of vaccines used against bovine mastitis

Antigens	Vaccine type	Effectiveness	Shortcomings	References
*Staphylococcus aureus*	Bacterin (whole cell inactivated)	Reduction of the progression of clinical symptoms Induction of a strong short-term immune response	Not prevent mastitis or reduce the incidence	[65–68]
*Escherichia coli* J5 *Staphylococcus aureus*	Whole cell inactivated. *S. aureus* expresses Slime Associated Antigenic Complex (SAAC)	Reduction of the severity of clinical signs and duration of intramammary infections	Not reduce the rate of intramammary infections	[59,60,62,63]
*Staphylococcus aureus* *Streptococcus agalactiae* *Streptococcus dysgalactiae* *Streptococcus uberis* *Streptococcus pyogenes* *Escherichia coli* *Corynebacterium pyogenes*	Whole cell inactivated	Not reported	Not reduce the incidence of clinical mastitis, not lessen the severity of symptoms	[59]
*Escherichia coli* J5	Whole cell inactivated	Reduction of the severity of infection	Not reduce rates of coliforms mastitis or all types of clinical mastitis	[61,69]
*Staphylococcus aureus* *Staphylococcus chromogenes*	Surface-associated proteins	Reduction in the incidence of staphylococcal mastitis.	Conferred partial protection from natural infection.	[70]
*Staphylococcus aureus* *Streptococcus agalactiae*	Whole cell inactivated	Increase the concentration of IgG in the milk of vaccinated cows	Not reduce rates of intramammary infections	[71]

IgG, immunoglobulin G.

**Table 2:** Antimicrobial properties of nanoparticles on pathogens causing bovine mastitis

Nanoparticles	Pathogen	Advantages	Disadvantages	References
Chitosan	*Staphylococcus aureus* Coagulase-negative *Staphylococcus* *Pseudomonas* spp.	Damage to the plasma membrane of the bacterium causes lysis. Inhibit the formation of biofilms of the bacteria Not toxic for bovine cell lines Not promote the production of proinflammatory cytokines	Not reported *in vivo* evaluations	[14,16]
ZnO	*Escherichia coli* *Klebsiella pneumoniae* *Staphylococcus aureus*	Bactericidal effect on *E. coli* and *K. pneumoniae*	Not exhibit a bactericidal effect on *S. aureus* Not reported *in vivo* evaluations	[13]
Au (commercial)	*E. coli* *Streptococcus uberis* *S. aureus Candida albicans* *Candida krusei*	Reported cytotoxicity against *S. aureus* in Wistar rats	Exhibit weak bactericidal activity The pathogenic strains developed strong resistance against this NPs	[12,79]
Ag (commercial)	*E. coli* *Streptococcus uberis* *S. aureus Candida albicans* *Candida krusei*	Highest bactericidal effect	Cytotoxicity in eukaryotic cells The pathogenic strains developed strong resistance against this NPs	[12,79–84]
Cu (commercial)	*E. coli* *Streptococcus uberis* *S. aureus Candida albicans* *Candida krusei*	Highest bactericidal effect	Cytotoxicity in eukaryotic cells	[11,78]
Pt (commercial)	*E. coli* *Streptococcus uberis* *S. aureus Candida albicans* *Candida krusei*	Not reported	Not biocidal effect	[12]
Ag and Cu reduced with plant extracts	*Staphylococcus aureus* *Escherichia coli* *Streptococcus agalactiae Streptococcus dysgalactiae* *Salmonella* spp. *Enterococcus faecalis* *Enterobacter cloacae* *Candida albicans*	Cytotoxic on pathogens Not have a cytotoxic effect on mammary gland tissue cell lines Inhibit the formation of biofilms	Not reported *in vivo* evaluations	[15,17]
CuO reduced with ginger extract	Multidrug-resistant *S. aureus*	Exhibit bactericidal activity	Not reported *in vivo* evaluations	[18]
CuO reduced with garlic extract	Multidrug-resistant *S. aureus*	Exhibit bactericidal activity	Not reported *in vivo* evaluations	[18]
AgNPs reduced with cinnamon oil	Multidrug-resistant *Streptococcus agalactiae*	Showed reasonable antimicrobial Inhibition of biofilm formation	Cinnamon oil alone had a better effect than AgNPs	[15]
BiNPs	*Campylobacter jejuni* *Listeria monocytogenes* *Yersinia enterocolitica* *Salmonella typhimurium* *E. coli* *Mycoplasma argini* *Acholeplasma ladidlawii* *Bacillus anthracis* *Leptospira Pomona* *S. aureus* *Candida albicans* *Poryphyromonas gingivalis* *Methicillin-resistant S. aureus* *Enterococcus faecalis* *Streptococcus gordonii*	Exhibit antimicrobial activity Reduce microbial biofilm formation	Not reported *in vivo* evaluations	[85–90]
Fe_2_O_3_ NPs	*Bacillus subtilis Bacillus licheniformis* *S. aureus* *Bacillus brevis Vibrio cholerae, Streptococcus aureus* *S.epidermidis* *E. coli* *Shigella flexneri Pseudomonas aeruginosa*	Inhibition of biofilm formation	They have not been tested on clinical mastitis isolates Conflicting results regarding the antibacterial effect	[91–93]

NPs, nanoparticles.
